# Amino acid digestibility in plant protein sources fed to growing pigs

**DOI:** 10.5713/ajas.19.0037

**Published:** 2019-03-07

**Authors:** Ah Reum Son, Chan Sol Park, Kyu Ree Park, Beob Gyun Kim

**Affiliations:** 1 Department of Animal Science and Technology, Konkuk University, Seoul 05029, Korea; 2 Monogastric Animal Feed Research Institute, Konkuk University, Seoul 05029, Korea

**Keywords:** Feedstuff, Protein Supplements, Standardized Ileal Digestibility, Swine

## Abstract

**Objective:**

The objective was to determine standardized ileal digestibility (SID) of amino acids (AA) in 11 plant protein sources fed to growing pigs.

**Methods:**

Eleven feed ingredients used were sesame meal, two sources of soybean meal (SBM) produced in the Republic of Korea, a source of SBM produced in India, high-protein distillers dried grains (HPDDG), perilla meal, canola meal, copra meal, corn germ meal, palm kernel expeller, and tapioca distillers dried grains (TDDG). Experimental diets were prepared to contain each test ingredient as a sole source of AA, and a nitrogen-free diet was also prepared to estimate the basal ileal endogenous losses of AA. Twelve barrows surgically fitted with T-cannulas at the distal ileum with an initial body weight of 29.0 kg (standard deviation = 3.0) were individually housed in metabolism crates equipped with a feeder and a nipple drinker. A 12×9 incomplete Latin square design was employed with 12 experimental diets, 12 animals, and 9 periods. After a 5-d adaptation period, ileal digesta were collected on d 6 and 7 in each experimental period.

**Results:**

Values for apparent ileal digestibility of most indispensable AA in three sources of SBM were greater compared with other test ingredients except HPDDG and canola meal (p<0.05). Pigs fed diets containing SBM sources had also greater SID of most indispensable AA compared with those fed diets containing other test ingredients (p<0.05) except for HPDDG and canola meal. There was no difference in the apparent ileal digestibility and SID of AA among sources of SBM. The TDDG had the least value for the SID of methionine among test ingredients (p<0.05).

**Conclusion:**

The SID of most AA in SBM, HPDDG, and canola meal were greater than those in sesame meal, perilla meal, copra meal, and TDDG.

## INTRODUCTION

Dietary supplementation of protein and amino acids (AA) is important to promote normal and optimal growth for pigs [1]. Soybean meal (SBM) is one of the most commonly used protein sources in swine diets. However, researchers and feed formulators have tried to find alternative feed ingredients to replace SBM due to its relatively high price [2]. Many plant protein sources produced from oil-extraction and distillation processes have been considered as alternative feed ingredients because crude protein (CP) and AA contents in grains, oilseeds, or fruit byproducts were concentrated after processing [3].

To use an alternative feed ingredient in swine diets, nutritional values of the ingredient should be considered [4]. The concentration of biologically available AA in a protein supplement is one of the most important factors in deciding the use of the protein supplement in swine diets. The bioavailability of AA for the pigs is generally expressed as a standardized ileal digestibility (SID) [5]. However, information on the SID of AA in some plant protein sources is very limited. Therefore, this experiment was conducted to determine the SID of AA in nine plant protein sources produced from the oil-extraction process and two plant protein sources produced from distillation process fed to growing pigs.

## MATERIALS AND METHODS

### Animal care

The experimental procedure was approved by the Institutional Animal Care and Use Committee at Konkuk University (KU12090).

### Ingredients and diets

Eleven plant protein sources used in the present study were identical to the ingredients reported by Son et al [6]. Test ingredients were sesame meal, two sources of dehulled SBM from Korea (SBM-KD 1 and SBM-KD 2), SBM from India (SBM-I), high-protein distillers dried grains produced from corn in the USA (HPDDG), perilla meal, canola meal (CM) from Indonesia, copra meal from the Philippines, corn germ meal (CGM), palm kernel expellers from Malaysia, and tapioca distillers dried grains from China (TDDG; Table 1). The copra and palm kernel byproducts were classified as meal and expellers, respectively, based on the concentration of ether extract in each ingredient [7].

Experimental diets were formulated to contain their respective test ingredients as a sole source of nitrogen (Tables 2, 3). A nitrogen-free diet was also prepared to estimate the basal ileal endogenous losses of AA. A 0.5% of chromic oxide was included in all experimental diets as an indigestible index. All experimental diets were formulated to contain adequate vitamins and minerals to meet or exceed the requirement estimates reported by the NRC [5].

### Animal, feeding, and sample collection

Twelve crossbred barrows with a mean initial body weight (BW) of 29.0±2.0 kg were surgically fitted with T-cannulas at the distal ileum based on the procedure described by Stein et al [8], and were individually placed in metabolism crates equipped with a feeder and a nipple drinker. The animals were allotted to a 12×9 incomplete Latin square design with 12 dietary treatments and 9 periods using a spreadsheet-based program to prevent potential carryover effects [9]. Based on the BW of each pig and metabolizable energy concentration of the experimental diets, daily feed allowance for each pig was calculated at the beginning of each experimental period as 2.7 times the estimated energy requirement for maintenance (i.e., 106 kcal of metabolizable energy per kg BW^0.75^). The amount of feed allowance was divided into two equal meals, and the feed was fed to pigs at 0800 and 1600 h. Water was available all the time. The BW of pigs was individually measured at the beginning of each period.

An experimental period consisted of a 5-d adaptation period and a 2-d collection period. The ileal digesta were collected from 0830 to 1600 h on d 6 and 7. For collecting the ileal digesta, a plastic bag was tied on the T-cannula using a wire, and the bag was changed every 30 min. The collected ileal digesta samples were immediately stored at −20°C.

### Chemical analysis

Ileal digesta samples were freeze-dried and finely ground before analyses. The AA concentrations in the ingredients, diets, and ileal digesta were analyzed using acid hydrolysis method (method 994.12) except for sulfur-containing AA (method 985.28) and tryptophan (method 988.15) [10]. For analyzing chromium concentration in the diet and ileal digesta, an ultraviolet-visible spectrophotometry (Optizen 2120UV, Mecasys Inc., Daejeon, Korea) was used.

### Calculation and statistical analysis

Values for apparent ileal digestibility (AID) and SID of AA were calculated based on the equations reported by a previous study [11]. Data were analyzed using MIXED procedure of SAS (SAS Inst. Inc., Cary, NC, USA). The model included dietary treatment as a fixed variable and animal and period as random variables [12]. Least squares means of each treatment were calculated and the difference among the least squares means was tested using the PDIFF option of SAS with the Tukey’s adjustment. The experimental unit was a pig, and the statistical significance was set at a p-value less than 0.05.

## RESULTS

All animals were maintained healthy and consumed provided experimental diets well. Values for the AID of most indispensable AA in three sources of SBM were greater compared with other test ingredients except HPDDG and CM (p<0.05; Table 4). There was no difference in the AID of AA among the sources of SBM. Pigs fed diets containing SBM-KD 1 and SBM-I had greater AID of lysine compared with other test ingredients (p<0.05) except for SBM-KD 2, which were not different from values in HPDDG and CM. Values for the AID of methionine in SBM-KD 2 and HPDDG were greater than in other test ingredients (p<0.05) except for SBM-KD 1, SBM-I, CM, and CGM. The AID of threonine and valine in SBM-KD 1 was greater than other test ingredients (p<0.05) but was not different from values in SBM-KD 2, SBM-I, and HPDDG. Pigs fed diets containing SBM sources and HPDDG had greater AID of isoleucine, leucine, and phenylalanine (p<0.05) except for those fed diet containing CM. Values for the AID of arginine, methionine, and phenylalanine in TDDG was the least among test ingredients (p<0.05).

Values for the SID of lysine in SBM-KD 1 and SBM-I were greater than other test ingredients (p<0.05) except for SBM-KD 2 and HPDDG, which were not different from values in CM (Table 5). Values for the SID of methionine in SBM-KD 1, SBM-KD 2, and HPDDG were greater than in sesame meal, perilla meal, copra meal, and TDDG (p<0.05) but were not different from values in SBM-I, CM, CGM, and palm kernel expellers. The TDDG had the lowest value for the SID of methionine among the test ingredients (p<0.05). Pigs fed diets containing SBM-KD1 had greater SID of threonine and valine compared with those fed diets containing other test ingredients (p<0.05) except for SBM-KD2, SBM-I, and HPDDG. Values for the SID of tryptophan in SBM-KD 1 and SBM-I were greater than perilla meal, copra meal, palm kernel expellers, and TDDG (p<0.05) but were not different from other test ingredients. Pigs fed diets containing SBM sources and HPDDG had greater SID of isoleucine, leucine, and phenylalanine compared with other test ingredients (p<0.05) except for those fed diet containing CM.

## DISCUSSION

The basal ileal endogenous losses of indispensable AA determined in the present study were within a range reported in the literature [13]. The concentration of CP and AA in sesame meal was within range of previous studies [5,14–16]. However, values for the SID of indispensable AA, especially lysine, in sesame meal were less than values in previous studies [5, 15,16]. The reason for this result may be due to different oil-extraction processes and conditions [16]. In addition, fiber concentration in the sesame meal used in the present study and Son et al [6] was greater compared with those in the previous studies [5,15,16]. Although the SID of most indispensable AA in sesame meal was less than in SBM sources used in the present study, sesame meal had greater concentrations of methionine and tryptophan compared with SBM and other test ingredients. Based on the present results, therefore, sesame meal can be used as a good source of methionine and tryptophan in swine diets in agreement with a previous work [16].

The AA composition in the three sources of SBM agreed with the tabular values in the literature [5,14]. However, the SID values of AA in the three SBM sources were a bit less than those in the literature [5,14]. The SID values of AA in SBM reported in other previous studies [17,18] were similar to those in the present work. During SBM production process, soybean is dehulled, and then soyhulls are often added to the dehulled SBM after oil-extraction process resulting in SBM with hulls [19]. The concentration of CP in SBM is affected by the inclusion rate of hulls, and the AA concentration is highly correlated with the CP concentration [19]. In the present work, the SBM-KD 1 and 2 had greater concentrations of CP and most AA compared with SBM-I that contained hulls. The AA digestibility can be decreased as the inclusion rate of hulls or the dietary fiber content increases [20,21]. However, we failed to find the differences in the SID of AA among the three SBM sources used in the present study, in agreement with Park et al [22] who reported no difference in the SID of AA between two SBM sources with varying soy hull inclusion rates. A potential reason for this discrepancy is that the three SBM sources used in this work had similar fiber concentrations [6] regardless of hull inclusion rates.

The CP and most AA concentrations of HPDDG used in the present work were within the range of previously reported values [5,23–25]. However, there was a relatively large variability in CP and AA concentrations in the literature. The variability perhaps is attributed to different dehulling, degerming, or both processes before fermentation for ethanol production. Values for the SID of most AA in HPDDG determined in the present study were within the range of previous values [5,23–25]. Among the test ingredients in this study, HPDDG had most comparable values for the AA digestibility with SBM sources. Due to the low lysine and tryptophan concentrations in HPDDG, however, the value and the potential inclusion rate of HPDDG in swine diets would be limited.

The AA concentration in CM used in this study agreed with previously reported values [5,26,27]. The SID of most AA in CM used in this study was less than those in the literature. The fiber concentrations in CM used in the present work did not deviate much from the literature [5,26–28]. A previous study comparing the AA digestibility among seven sources of CM reported that the differences of 5% to 10% units were observed among the CM sources. This difference may have resulted from different genotypes of seeds and oil-extraction conditions [27,28].

The CP and AA concentrations in perilla meal, copra meal, CGM, and palm kernel expellers used in the present study agreed with values in the literature [5,11,29–31]. However, values for the SID of most indispensable AA in the test ingredients were less compared with those in the literature [5, 11,29–31]. It remains unclear why the SID of AA determined in this study were less than in the previously reported values. However, it has been reported that the digestibility of AA in the feed ingredients produced from the oil-extraction process can be affected by several factors including drying condition, heat damage, regional origins of grains or oilseeds, and species [27,31,32]. To our knowledge, the digestibility of AA in TDDG fed to the pigs has not been reported. The TDDG had less digestibility of most AA compared with other test ingredients used in this study. The reason for this result may be due to the high concentration of neutral detergent fiber and acid detergent fiber in TDDG [6,11].

In the present study, the AA digestibility of lysine was less than other indispensable AA in most test ingredients. In the oil-extraction and distillation processes, heating and drying are essential steps, and thus, byproducts can be damaged by heat. It has been reported that lysine is the most influenced by heat damage associated with Maillard reaction during the thermal processing [32]. In addition, anti-nutritional factors such as trypsin inhibitors, glucosinolates, and β-mannans in the plant protein sources may negatively affect AA digestibility [26,33]. The lack of analysis for anti-nutritional factors in the test ingredients is a limitation of the present study. Further research is warranted to quantify the influence of anti-nutritional factors on AA digestibility.

In conclusion, sources of SBM used in the present study had greater values for the SID of most AAs compared with other test ingredients. Although the HPDDG used in this study had high AA digestibility values comparable to the AA digestibility of SBM, digestible lysine and tryptophan concentrations in the HPDDG with solubles were less than those in SBM. Swine feed producers can use the data provided in the present work with combination with other nutrient concentrations and prices in determining the value of each ingredient.

## Figures and Tables

**Table 1:** Energy and nutrient composition of test ingredients (as-is basis)

Item1)	Ingredient										

	Sesame meal	Soybean meal-dehulled-Korea 1	Soybean meal-dehulled-Korea 2	Soybean meal-India	High-protein distillers dried grains	Perilla meal	Canola meal	Copra meal	Corn germ meal	Palm kernel expellers	Tapioca distillers dried grains
Indispensable amino acid (%)											
Arginine	3.79	3.46	3.51	2.97	1.32	3.87	2.40	1.65	1.42	1.62	0.66
Histidine	1.17	1.23	1.29	1.07	1.10	1.09	0.99	0.43	0.69	0.31	0.38
Isoleucine	1.74	2.18	2.26	1.78	1.60	1.56	1.31	0.66	0.76	0.52	0.88
Leucine	3.32	3.79	3.88	3.19	5.70	2.88	2.48	1.37	1.74	1.05	1.37
Lysine	1.02	3.15	3.30	2.68	1.23	1.15	1.76	0.51	1.00	0.48	0.96
Methionine	1.05	0.31	0.48	0.34	0.52	0.54	0.47	0.16	0.23	0.18	0.14
Phenylalanine	2.50	2.42	2.49	2.23	2.39	2.48	1.51	1.09	1.03	0.82	0.82
Threonine	1.39	2.00	2.01	1.67	1.45	1.35	1.50	0.69	0.90	0.53	0.80
Tryptophan	0.63	0.49	0.51	0.42	0.22	0.43	0.41	0.14	0.19	0.09	0.13
Valine	2.01	2.10	2.16	1.75	1.79	1.85	1.61	0.95	1.08	0.74	0.98
Dispensable amino acid (%)											
Alanine	2.38	2.10	2.14	1.77	3.05	2.02	1.61	0.92	1.29	0.69	1.00
Aspartic acid	3.38	5.54	5.61	4.53	2.42	3.21	2.42	1.63	1.67	1.27	1.57
Cysteine	0.21	0.61	0.79	0.56	0.52	0.26	0.97	0.29	0.34	0.19	0.14
Glutamic acid	8.81	8.61	8.70	7.48	7.22	7.70	6.59	3.84	3.25	2.96	1.87
Glycine	2.35	2.05	2.09	1.71	1.29	2.02	1.85	0.92	1.18	0.74	0.79
Proline	1.06	1.82	1.76	1.60	3.11	0.80	1.64	0.49	0.99	0.35	0.55
Serine	1.29	2.40	2.40	2.06	1.85	1.51	1.53	0.84	1.05	0.68	0.75
Tyrosine	1.77	1.66	1.47	1.48	1.60	1.53	1.04	0.54	0.68	0.46	0.50
Dry matter (%)	97.0	90.2	90.2	90.1	91.5	90.3	91.4	90.2	94.1	89.6	93.3
Gross energy (kcal/kg)	4,688	4,299	4,332	4,221	4,924	4,240	4,235	4,095	4,699	4,407	3,875
Crude protein (%)	50.0	47.1	47.4	39.6	38.0	43.2	37.5	21.8	21.4	15.3	18.4
Ether extract (%)	6.05	2.46	0.74	0.84	5.24	1.08	1.85	1.76	8.27	6.97	3.12
Crude fiber (%)	9.3	4.6	5.7	5.1	7.3	18.8	9.6	13.6	10.4	17.0	22.7
Ash (%)	11.0	6.2	6.3	6.3	1.4	9.0	9.5	6.7	2.4	4.7	14.9
Neutral detergent fiber (%)	28.1	7.4	8.7	9.6	39.0	44.7	24.7	55.1	43.4	61.4	56.2
Acid detergent fiber (%)	17.5	7.2	9.1	8.2	20.1	25.9	18.1	32.2	14.6	36.8	47.3
Calcium (%)	2.15	0.64	0.67	0.70	0.13	1.71	1.01	0.62	0.13	0.43	0.77
Phosphorus (%)	1.32	0.64	0.62	0.53	0.25	1.25	0.95	0.54	0.53	0.55	0.22

1) The analyzed energy and nutrient compositions except amino acids are adapted from Son et al [6].

**Table 2:** Ingredient and chemical composition of experimental diets (as-fed basis)

Items	Diet											

	Sesame meal	Soybean meal-dehulled-Korea 1	Soybean meal-dehulled-Korea 2	Soybean meal-India	High-protein distillers dried grains	Perilla meal	Canola meal	Copra meal	Corn germ meal	Palm kernel expellers	Tapioca distillers dried grains	Nitrogen-free
Ingredient (%)												
Corn starch	48.6	44.2	42.2	43.3	36.7	38.6	38.3	37.0	36.8	37.3	37.0	68.4
Sucrose	20.0	20.0	20.0	20.0	20.0	20.0	20.0	20.0	20.0	20.0	20.0	20.0
Sesame meal	30.0	-	-	-	-	-	-	-	-	-	-	-
Soybean meal-dehulled-Korea 1	-	33.0	-	-	-	-	-	-	-	-	-	-
Soybean meal-dehulled-Korea 2	-	-	35.0	-	-	-	-	-	-	-	-	-
Soybean meal-India	-	-	-	34.0	-	-	-	-	-	-	-	-
High-protein distillers dried grains	-	-	-	-	40.0	-	-	-	-	-	-	-
Perilla meal	-	-	-	-	-	40.0	-	-	-	-	-	-
Canola meal	-	-	-	-	-	-	40.0	-	-	-	-	-
Copra meal	-	-	-	-	-	-	-	40.0	-	-	-	-
Corn germ meal	-	-	-	-	-	-	-	-	40.0	-	-	-
Palm kernel expellers	-	-	-	-	-	-	-	-	-	40.0	-	-
Tapioca distillers dried grains	-	-	-	-	-	-	-	-	-	-	40.0	-
Soybean oil	-	-	-	-	-	-	-	-	-	-	-	4.00
Cellulose	-	-	-	-	-	-	-	-	-	-	-	4.00
Potassium carbonate	-	-	-	-	-	-	-	-	-	-	-	0.40
Magnesium oxide	-	-	-	-	-	-	-	-	-	-	-	0.10
Limestone	-	0.35	0.35	0.10	0.30	-	0.05	0.50	0.45	0.20	-	0.75
Dicalcium phosphate	-	1.10	1.10	1.25	1.65	-	0.25	1.15	1.35	1.15	1.60	1.00
Salt	0.40	0.40	0.40	0.40	0.40	0.40	0.40	0.40	0.40	0.40	0.40	0.40
Vitamin-mineral premix1)	0.50	0.50	0.50	0.50	0.50	0.50	0.50	0.50	0.50	0.50	0.50	0.50
Chromic oxide	0.50	0.50	0.50	0.50	0.50	0.50	0.50	0.50	0.50	0.50	0.50	0.50
Analyzed composition (%)												
Dry matter	93.7	91.2	90.9	91.5	92.1	91.3	91.9	91.5	92.7	91.0	92.5	92.0
Crude protein	16.0	19.0	18.7	13.6	14.2	18.1	14.7	8.28	8.13	6.10	7.71	0.27
Ether extract	1.73	0.46	0.55	0.53	1.49	0.31	0.76	0.84	3.34	2.61	0.48	2.10
Ash	4.30	4.45	4.62	4.50	3.72	5.04	5.29	4.96	3.27	4.08	9.08	3.07

1) Provided the following quantities per kg of complete diet: vitamin A, 25,000 IU; vitamin D_3_, 4,000 IU; vitamin E, 50 IU; vitamin K, 5.0 mg; thiamin, 4.9 mg; riboflavin, 10.0 mg; pyridoxine, 4.9 mg; vitamin B_12_, 0.06 mg; pantothenic acid, 37.5 mg; folic acid, 1.10 mg; niacin, 62 mg; biotin, 0.06 mg; Cu, 25 mg as copper sulfate; Fe, 268 mg as iron sulfate; I, 5.0 mg as potassium iodate; Mn, 125 mg as manganese sulfate; Se, 0.38 mg as sodium selenite; Zn, 313 mg as zinc oxide; and butylated hydroxytoluene, 50 mg.

**Table 3:** Amino acids (AA) concentration of experimental diets (%, as-fed basis)

Items	Diet										

	Sesame meal	Soybean meal-dehulled-Korea 1	Soybean meal-dehulled-Korea 2	Soybean meal-India	High-protein distillers dried grains	Perilla meal	Canola meal	Copra meal	Corn germ meal	Palm kernel expellers	Tapioca distillers dried grains
Indispensable AA											
Arginine	0.95	1.23	0.94	1.09	0.55	0.52	1.00	0.64	0.53	0.76	0.18
Histidine	0.31	0.39	0.38	0.35	0.45	0.43	0.43	0.18	0.28	0.14	0.11
Isoleucine	0.49	0.63	0.68	0.55	0.68	0.64	0.61	0.29	0.31	0.26	0.24
Leucine	0.90	1.16	1.16	1.02	2.37	2.25	1.09	0.59	0.68	0.50	0.37
Lysine	0.23	0.30	0.92	0.27	0.47	0.44	0.72	0.21	0.36	0.22	0.28
Methionine	0.38	0.37	0.20	0.18	0.36	0.35	0.24	0.09	0.14	0.11	0.07
Phenylalanine	0.64	0.82	0.76	0.73	0.91	0.86	0.65	0.44	0.40	0.38	0.24
Threonine	0.36	0.46	0.57	0.41	0.60	0.57	0.60	0.29	0.34	0.25	0.23
Tryptophan	0.15	0.25	0.15	0.10	0.09	0.18	0.16	0.06	0.07	0.04	0.04
Valine	0.59	0.75	0.66	0.67	0.77	0.73	0.72	0.44	0.48	0.39	0.30
Dispensable AA											
Alanine	0.64	0.83	0.63	0.73	1.27	1.21	0.67	0.40	0.51	0.33	0.29
Aspartic acid	0.91	1.16	1.61	1.03	1.00	0.95	1.01	0.69	0.64	0.61	0.45
Cysteine	0.11	0.05	0.29	0.21	0.39	0.19	0.41	0.15	0.17	0.11	0.07
Glutamic acid	2.42	3.07	2.55	2.71	2.97	2.82	2.74	1.67	1.27	1.43	0.55
Glycine	0.63	0.81	0.61	0.72	0.55	0.52	0.77	0.40	0.47	0.34	0.23
Proline	0.30	0.37	0.53	0.33	1.30	1.23	0.73	0.21	0.36	0.16	0.14
Serine	0.32	0.40	0.68	0.36	0.74	0.71	0.59	0.34	0.38	0.30	0.23
Tyrosine	0.36	0.52	0.37	0.46	0.57	0.54	0.40	0.16	0.20	0.17	0.12

**Table 4:** Apparent ileal digestibility (%) of amino acids (AA) in 11 sources of plant protein sources fed to pigs

Items	Diet											SEM	p-value

	Sesame meal	Soybean meal-dehulled-Korea 1	Soybean meal-dehulled-Korea 2	Soybean meal-India	High-protein distillers dried grains	Perilla meal	Canola meal	Copra meal	Corn germ meal	Palm kernel expellers	Tapioca distillers dried grains		
No. of observation	7	8	7	8	7	8	7	8	6	6	6	-	-
Indispensable AA													
Arginine	59.5^de^	90.4^a^	85.7^ab^	86.6^ab^	70.4^cd^	65.5^cd^	73.5^bc^	48.7^e^	69.2^cd^	61.9cde	24.3^f^	3.2	<0.001
Histidine	45.7^cd^	85.5^a^	80.9^ab^	82.6^a^	74.8^ab^	48.2^cd^	72.6^ab^	40.7^d^	64.3^bc^	45.9^cd^	30.7^d^	4.0	<0.001
Isoleucine	37.3^c^	79.9^a^	76.3^a^	76.7^a^	74.2^a^	40.5^c^	65.1^ab^	36.8^c^	44.9^c^	48.6^bc^	29.0^c^	4.0	<0.001
Leucine	46.5^c^	81.6^a^	77.8^a^	78.7^a^	84.5^a^	46.1^c^	69.8^ab^	45.5^cd^	58.4^bc^	53.9^c^	30.7^d^	3.4	<0.001
Lysine	7.0^ef^	84.1^a^	78.0^ab^	82.1^a^	59.8^bc^	26.4^de^	57.5^bc^	7.4^f^	43.6^cd^	32.7^d^	35.7^d^	4.8	<0.001
Methionine	61.7^bc^	79.0^ab^	86.9^a^	77.9^ab^	89.0^a^	34.0^d^	72.8^ab^	45.1^cd^	66.9abc	59.1^bc^	4.5^e^	5.5	<0.001
Phenylalanine	51.8^c^	82.3^a^	79.3^a^	81.3^a^	79.5^a^	55.2^c^	69.8^ab^	52.8^c^	57.7^bc^	57.3^bc^	31.5^d^	3.1	<0.001
Threonine	17.9^d^	73.8^a^	65.0^ab^	69.0^ab^	63.9^ab^	27.8^d^	52.0^bc^	20.1^d^	31.6^cd^	30.0^d^	11.4^d^	4.8	<0.001
Tryptophan	66.6abc	82.4^a^	79.6^a^	82.3^a^	71.6^ab^	55.8^bc^	62.2abc	45.0^cd^	58.8abc	41.3^cd^	23.2^d^	5.9	<0.001
Valine	31.1^de^	73.9^a^	69.2^ab^	68.9^ab^	68.8^ab^	34.9^de^	58.6^bc^	38.1^d^	47.4^cd^	46.1^cd^	19.4^e^	3.8	<0.001
Dispensable AA													
Alanine	33.6^de^	72.5^ab^	66.1abc	67.7^ab^	78.1^a^	30.4^de^	58.0^bc^	30.2^de^	46.6^cd^	37.1^de^	20.9^e^	4.6	<0.001
Aspartic acid	16.6^d^	79.7^a^	72.6^ab^	77.5^a^	63.9^ab^	24.1^d^	51.1^bc^	27.3^d^	30.7^cd^	33.9^cd^	17.2^d^	5.3	<0.001
Cysteine	−81.4^d^	63.1^a^	68.3^a^	60.2^a^	70.6^a^	−32.1^c^	61.7^a^	11.4^b^	35.6^ab^	19.9^b^	−54.6^cd^	8.0	<0.001
Glutamic acid	39.8^b^	84.9^a^	79.2^a^	81.6^a^	79.5^a^	43.0^b^	76.0^a^	43.1^b^	57.0^b^	49.8^b^	20.3^c^	4.3	<0.001
Glycine	−5.0^de^	63.6^a^	50.8^a^	46.6^ab^	38.8abc	10.8^cd^	37.5abc	−3.0^de^	12.9bcde	−19.1^de^	−24.8^e^	7.9	<0.001
Proline	−248.7^bc^	39.7^a^	−2.0^ab^	−36.6abc	38.2^a^	−241.6^bc^	−25.9abc	−280.0^c^	−146.3abc	−631.9^d^	−208.1abc	62.0	<0.001
Serine	21.1^de^	79.8^a^	71.5^ab^	75.8^ab^	72.9^ab^	37.8^cd^	62.0^b^	31.7^cd^	41.2^c^	38.7^cd^	8.2^e^	4.5	<0.001
Tyrosine	55.7cde	83.4^a^	72.1abc	81.9^ab^	78.6^ab^	54.2^de^	65.5bcd	29.7^f^	38.4^ef^	42.9^ef^	35.2^f^	3.8	<0.001

SEM, standard error of the mean.

a–f Means within a row without a common superscript differ (p<0.05).

**Table 5:** Standardized ileal digestibility (%) of amino acids (AA) in 11 sources of plant protein sources fed to pigs [1]

Items	Diet											SEM	p-value

	Sesame meal	Soybean meal-dehulled-Korea 1	Soybean meal-dehulled-Korea 2	Soybean meal-India	High-protein distillers dried grains	Perilla meal	Canola meal	Copra meal	Corn germ meal	Palm kernel expellers	Tapioca distillers dried grains		
No. of observation	7	8	7	8	7	8	7	8	6	6	6	-	-
Indispensable AA													
Arginine	64.3^d^	95.2^a^	91.5^ab^	92.0^ab^	80.4^bc^	69.1^cd^	79.0^bc^	57.3^de^	79.6^bc^	69.1^cd^	45.1^e^	3.2	<0.001
Histidine	49.9^d^	89.2^a^	84.7^ab^	86.6^ab^	78.1^ab^	51.6^cd^	76.0^ab^	48.9^d^	69.6^bc^	56.4^cd^	40.4^d^	4.0	<0.001
Isoleucine	41.8^cd^	83.1^a^	79.8^a^	80.6^a^	77.7^a^	44.3^cd^	68.9^ab^	44.8^cd^	52.4bcd	57.6^bc^	35.7^d^	4.0	<0.001
Leucine	50.2^cd^	84.6^a^	81.1^a^	82.2^a^	86.1^a^	49.4^cd^	73.3^ab^	51.9^cd^	63.9^bc^	61.4^bc^	37.5^d^	3.4	<0.001
Lysine	16.6^g^	86.9^a^	81.2abc	85.3^ab^	66.1bcd	32.8^fg^	61.6cde	21.4^g^	51.8def	46.0def	43.3^ef^	4.8	<0.001
Methionine	64.6^bc^	87.9^a^	91.5^a^	85.8^ab^	91.5^a^	38.3^de^	76.6abc	55.2^cd^	73.4abc	67.4abc	20.8^e^	5.5	<0.001
Phenylalanine	55.4^c^	85.7^a^	82.8^a^	84.9^a^	82.4^a^	57.9^c^	73.9^ab^	58.9^c^	64.4^bc^	64.4^bc^	39.6^d^	3.1	<0.001
Threonine	29.2^de^	80.9^a^	73.3^ab^	77.3^ab^	71.7^ab^	36.5^de^	59.8^bc^	36.3^de^	45.5cde	48.9^cd^	26.1^e^	4.8	<0.001
Tryptophan	70.8abc	87.3^a^	84.9^ab^	87.9^a^	80.4abc	60.4^cd^	67.1abc	58.1^cd^	70.1abc	61.1bcd	38.4^d^	5.9	<0.001
Valine	38.8^fg^	80.5^a^	76.2^ab^	76.7^ab^	74.8^ab^	41.1efg	65.0^bc^	48.6def	57.0cde	57.9^cd^	31.2^g^	3.8	<0.001
Dispensable AA													
Alanine	40.8^de^	79.9^a^	74.3^ab^	76.2^a^	82.1^a^	36.8^e^	65.7abc	43.0^de^	56.6bcd	52.7cde	33.7^e^	4.6	<0.001
Aspartic acid	22.9^d^	83.1^a^	76.6^ab^	81.6^a^	70.2^ab^	29.0^d^	57.4^bc^	36.5^cd^	40.6^cd^	44.3^cd^	27.3^d^	5.3	<0.001
Cysteine	−46.8^e^	74.0^ab^	75.8^a^	71.7^ab^	76.2^a^	−11.1^d^	67.0^ab^	26.0^c^	48.4abc	39.7^bc^	−15.7^de^	8.0	<0.001
Glutamic acid	42.6^de^	87.6^a^	82.2^a^	84.6^a^	82.0^a^	45.5cde	78.8^ab^	47.6cde	62.9^bc^	55.1^cd^	30.4^e^	4.3	<0.001
Glycine	14.9^e^	84.4^a^	73.8^ab^	70.8^ab^	64.4abc	28.2^de^	55.7abcd	32.2cde	42.9bcde	22.3^de^	19.7^de^	7.9	<0.001
Proline	−64.1^ab^	137.4^a^	108.8^a^	71.4^a^	83.4^a^	−58.2^ab^	54.5^a^	−0.5^ab^	16.7^ab^	−265.0^b^	58.8^a^	62.0	<0.001
Serine	33.4^de^	85.8^a^	78.5^a^	82.6^a^	79.3^a^	45.7^cd^	70.1^ab^	45.7^cd^	53.7^bc^	54.6^bc^	24.1^e^	4.5	<0.001
Tyrosine	59.1^cd^	86.7^a^	77.0^ab^	85.5^ab^	81.7^ab^	57.1^cd^	70.1^bc^	41.0^e^	47.4^de^	53.6cde	44.3^de^	3.8	<0.001

SEM, standard error of the mean.

1) Values for the standardized ileal digestibility of amino acids were calculated by correcting the apparent ileal digestibility for basal endogenous losses. The basal endogenous losses (g/kg dry matter intake) were determined from pigs fed an nitrogen-free diet: arginine, 0.60; histidine, 0.16; isoleucine, 0.25; leucine, 0.41; lysine, 0.32; methionine, 0.10; phenylalanine, 0.29; threonine, 0.51; tryptophan, 0.09; valine, 0.50; alanine, 0.56; aspartic acid, 0.69; cysteine, 0.24; glutamic acid, 0.82; glycine, 1.53; proline, 6.38; serine, 0.52; tyrosine, 0.20.

a–g Means within a row without a common superscript differ (p<0.05).

## References

[1] Lewis AJ, Lewis AJ, Southern LL (2001). Amino acids in swine nutrition. Swine nutrition.

[2] Woyengo TA, Beltranena E, Zijlstra RT (2014). Controlling feed cost by including alternative ingredients into pig diets: a review. J Anim Sci.

[3] Messad F, Létourneau-Montminy MP, Charbonneau E, Sauvant D, Guay F (2016). Meta-analysis of the amino acid digestibility of oilseed meal in growing pigs. Animal.

[4] Kiarie E, Nyachoti CM Alternative feed ingredients in swine diets.

[5] Committee on Nutrient Requirements of Swine, National Research Council (2012). Nutrient requirements of swine.

[6] Son AR, Park CS, Kim BG (2017). Determination and prediction of digestible and metabolizable energy concentrations in byproduct feed ingredients fed to growing pigs. Asian-Australas J Anim Sci.

[7] Lee SA, Kim BG (2017). Classification of copra meal and copra expellers based on ether extract concentration and prediction of energy concentrations in copra byproducts. J Anim Plant Sci.

[8] Stein HH, Shipley CF, Easter RA (1998). Technical note: A technique for inserting a T-cannula into the distal ileum of pregnant sows. J Anim Sci.

[9] Kim BG, Kim T (2010). A program for making completely balanced Latin Square designs employing a systemic method. Rev Colomb Cienc Pecu.

[10] Horwitz W, Latimer GW (2005). AOAC International Official methods of analysis of AOAC International.

[11] Son AR, Hyun Y, Htoo JK, Kim BG (2014). Amino acid digestibility in copra expellers and palm kernel expellers by growing pigs. Anim Feed Sci Technol.

[12] Seo S, Jeon S, Ha JK (2018). Guidelines for experimental design and statistical analyses in animal studies submitted for publication in the Asian-Australasian Journal of Animal Sciences. Asian-Australas J Anim Sci.

[13] Park CS, Oh SI, Kim BG (2013). Prediction of basal endogenous losses of amino acids based on body weight and feed intake in pigs fed nitrogen-free diets. Rev Colomb Cienc Pecu.

[14] Sauvant D, Perez JM, Tran G (2004). Tables of composition and nutritional value of feed materials: pigs, poultry, sheep, goats, rabbits, horses, and fish.

[15] Aguilera A, de Souza TCR, Mariscal-Landin G, Escobar K, Montaño S, Bernal MG (2015). Standardized ileal digestibility of proteins and amino acids in sesame expeller and soya bean meal in weaning piglets. J Anim Physiol Anim Nutr.

[16] Casas GA, Jaworski NW, Htoo JK, Stein HH (2018). Ileal digestibility of amino acids in selected feed ingredients fed to young growing pigs. J Anim Sci.

[17] Adebiyi AO, Ragland D, Adeola O, Olukosi OA (2015). Apparent or standardized ileal digestibility of amino acids of diets containing different protein feedstuffs fed at two crude protein levels for growing pigs. Asian-Australas J Anim Sci.

[18] Upadhaya SD, Kim IH (2015). Ileal digestibility of nutrients and amino acids in unfermented, fermented soybean meal and canola meal for weaning pigs. Anim Sci J.

[19] Lee SA, Park CS, Nam DS, Kim BG (2016). Prediction models for amino acid concentrations in soybean meal.

[20] Dilger RN, Sands JS, Ragland D, Adeola O (2004). Digestibility of nitrogen and amino acids in soybean meal with added soyhulls. J Anim Sci.

[21] Wang HL, Shi M, Xu X, Ma XK, Liu L, Piao XS (2017). Comparative energy content and amino acid digestibility of barley obtained from diverse sources fed to growing pigs. Asian-Australas J Anim Sci.

[22] Park CS, Helmbrecht A, Htoo JK, Adeola O (2017). Comparison of amino acid digestibility in full-fat soybean, two soybean meals, and peanut flour between broiler chickens and growing pigs. J Anim Sci.

[23] Kim BG, Petersen GI, Hinson RB, Allee GL, Stein HH (2009). Amino acid digestibility and energy concentration in a novel source of high-protein distillers dried grains and their effects on growth performance of pigs. J Anim Sci.

[24] Adeola O, Ragland D (2012). Ileal digestibility of amino acids in coproducts of corn processing into ethanol for pigs. J Anim Sci.

[25] Xue PC, Dong B, Zang JJ, Zhu ZP, Gong LM (2012). Energy and standardized ileal amino acid digestibilities of chinese distillers dried grains, produced from different regions and grains fed to growing pigs. Asian-Australas J Anim Sci.

[26] Liu Y, Song M, Maison T, Stein HH (2014). Effects of protein concentration and heat treatment on concentration of digestible and metabolizable energy and on amino acid digestibility in four sources of canola meal fed to growing pigs. J Anim Sci.

[27] Maison T, Stein HH (2014). Digestibility by growing pigs of amino acids in canola meal from North America and 00-rapeseed meal and 00-rapeseed expellers from Europe. J Anim Sci.

[28] Adewole DI, Rogiewicz A, Dyck B, Slominski BA (2016). Chemical and nutritive characteristics of canola meal from Canadian processing facilities. Anim Feed Sci Technol.

[29] Moon HK, Kim JW, Heo KN (1994). Growth performance and amino acid digestibilities affected by various plant protein sources in growing-finishing pigs. Asian-Australas J Anim Sci.

[30] Almeida FN, Petersen GI, Stein HH (2011). Digestibility of amino acids in corn, corn coproducts, and bakery meal fed to growing pigs. J Anim Sci.

[31] Stein HH, Casas GA, Abelilla JJ, Liu Y, Sulabo RC (2015). Nutritional value of high fiber co-products from the copra, palm kernel, and rice industries in diets fed to pigs. J Anim Sci Biotechnol.

[32] Kim BG, Kil DY, Zhang Y, Stein HH (2012). Concentrations of analyzed or reactive lysine, but not crude protein, may predict the concentration of digestible lysine in distillers dried grains with solubles fed to pigs. J Anim Sci.

[33] Cotten B, Ragland D, Thomson JE, Adeola O (2016). Amino acid digestibility of plant protein feed ingredients for growing pigs. J Anim Sci.

